# Characterization of the Bacteriophage BUCT603 and Therapeutic Potential Evaluation Against Drug-Resistant *Stenotrophomonas maltophilia* in a Mouse Model

**DOI:** 10.3389/fmicb.2022.906961

**Published:** 2022-07-05

**Authors:** Pengjun Han, Wenjing Zhang, Mingfang Pu, Yahao Li, Lihua Song, Xiaoping An, Mengzhe Li, Fei Li, Shuyan Zhang, Huahao Fan, Yigang Tong

**Affiliations:** ^1^College of Life Science and Technology, Beijing University of Chemical Technology, Beijing, China; ^2^School of Public Health, Lanzhou University, Lanzhou, China; ^3^Beijing Advanced Innovation Center for Soft Matter Science and Engineering, Beijing University of Chemical Technology, Beijing, China; ^4^Clinical Laboratory Center, Taian City Central Hospital, Taian, China; ^5^Department of Medical Technology Support, Jingdong Medical District of Chinese PLA General Hospital, Beijing, China

**Keywords:** *Stenotrophomonas maltophilia*, bacteriophage BUCT603, adsorption receptor, genomic analysis, structural protein, phage therapy

## Abstract

*Stenotrophomonas maltophilia* (*S. maltophilia*) is a common opportunistic pathogen that is resistant to many antibiotics. Bacteriophages are considered to be an effective alternative to antibiotics for the treatment of drug-resistant bacterial infections. In this study, we isolated and characterized a phage, BUCT603, infecting drug-resistant *S. maltophilia*. Genome sequencing showed BUCT603 genome was composed of 44,912 bp (32.5% G + C content) with 64 predicted open reading frames (ORFs), whereas no virulence-related genes, antibiotic-resistant genes or tRNA were identified. Whole-genome alignments showed BUCT603 shared 1% homology with other phages in the National Center for Biotechnology Information (NCBI) database, and a phylogenetic analysis indicated BUCT603 can be classified as a new member of the *Siphoviridae* family. Bacteriophage BUCT603 infected 10 of 15 *S. maltophilia* and used the TonB protein as an adsorption receptor. BUCT603 also inhibited the growth of the host bacterium within 1 h *in vitro* and effectively increased the survival rate of infected mice in a mouse model. These findings suggest that bacteriophage BUCT603 has potential for development as a candidate treatment of *S. maltophilia* infection.

## Introduction

*Stenotrophomonas maltophilia* (*S. maltophilia*) is an aerobic, non-fermenting, Gram-negative bacillus generally isolated from environmental sources, such as soil and water (Welker et al., 2015). *Stenotrophomonas maltophilia* is recognized as an important global opportunistic nosocomial pathogen, causing infections with considerable morbidity in immunocompromised patients (Looney et al., 2009; Esposito et al., 2017). Indeed, *S. maltophilia* can cause bacteremia, urinary tract infections, respiratory tract infections, skin and soft tissue infections (Abbott et al., 2011; Garazi et al., 2012). In recent years, the prevalence of *S. maltophilia* infection has increased due to irresponsible use of antibiotics and the increased placement of catheters and devices in patients, all of which are risk factors for *S. maltophilia* infection (Brooke, 2012; Osawa et al., 2018). More worryingly, *S. maltophilia* is intrinsically resistant to a number of antimicrobial classes, including most β-lactam antibiotics, cephalosporins, aminoglycosides and macrolides (Chang et al., 2015). Furthermore, these pathogenic bacteria form biofilms, making infections particularly difficult to treat clinically (Flores-Treviño et al., 2019). Trimethoprim–sulfamethoxazole (TMP–SMZ) has traditionally been considered as the first-line agent for treating *S. maltophilia* infections, but increasing reports of resistance to it and adverse drug effects and the lack of robust pharmacokinetic–pharmacodynamic data have limited its clinical usefulness (Toleman et al., 2007; Farrell et al., 2010; Hand et al., 2016; Ko et al., 2019). Fluoroquinolones have been considered an alternative treatment for *S. maltophilia* infection (Cho et al., 2014), but their role has also been limited by baseline and treatment-emergent resistance (Jia et al., 2015). The increasing lack of antimicrobials with activity against *S. maltophilia* is of great concern, so there is an urgent need to develop alternative methods for the treatment of these infections.

Bacteriophages (or phages) are more numerous and diverse than any other microbial entity (Díaz-Muñoz and Koskella, 2014). Over the past century, bacteriophages have been used for medical, food, agricultural, and ecological applications (Salmond and Fineran, 2015; Abedon and Katsaounis, 2018). In recent decades, with the rise of drug-resistant bacteria, bacteriophages have been regarded as one of the most promising alternatives to antibiotics for controlling antibiotic-resistant pathogenic bacteria (Torres-Barceló, 2018). Moreover, the cost of phage production is relatively lower than the development of novel antibiotics, and phage therapy is a green and efficient method for the treatment of drug-resistant bacteria because phage sources are extensive and their host specificity is high (Matsuzaki et al., 2005; Luong et al., 2020). Bacteriophages have allowed remarkable advances to be made in the prevention and treatment of bacterial infections, and have been successfully used to treat infections of the oral cavity, lung, reproductive tract, intestinal tract, heart, and skin (Kortright et al., 2019; Rehman et al., 2019; Melo et al., 2020). Therefore, phage therapy may be a reasonable choice for the prevention and treatment of drug-resistant *S. maltophilia* infections.

As we all know, bacteriophages are bacterial strains specific, so it will be necessary to establish a bacteriophage library containing a large number of phages specific for different bacterial hosts (Lin et al., 2017). Moreover, to ensure the safety of phages in clinical applications, the backgrounds of the phages used in therapy must be known precisely, to ensure the absence of toxins, resistance genes, virulence determinants, or a capacity to integrate into the host genome (temperate phages; McCallin et al., 2013; Clarke et al., 2020). Therefore, the characterization of newly isolated phages not only expands our knowledge of the biodiversity of bacteriophages, but also facilitates the development of phage therapy. In this study, we isolated and characterized a novel bacteriophage infecting drug-resistant *S. maltophilia*. An electron microscopic analysis of the virion, analysis of its molecular properties, a host range analysis, a full genome sequence analysis, a mass spectrometric analysis of the phage proteins, and evaluations of its therapeutic efficacy *in vitro* and *in vivo* were performed to comprehensively understand the antimicrobial potential of virulent *S. maltophilia* phages.

## Materials and Methods

### Bacterial Strains, Plasmid, Bacteriophage Isolation, and Culture Conditions

All clinical isolates of *S. maltophilia* used in this study were antibiotic-resistant strains and stored at −80°C in our lab. *Stenotrophomonas maltophilia* ATCC13637 and pBBR1MCS1 plasmid were kindly provided by Dr. Fangfang Wang, Institute of Microbiology, Chinese Academy of Sciences. All strains were cultivated in Luria–Bertani (LB) medium at 37°C. *Stenotrophomonas maltophilia* isolate 118 (SMA118) was used as a host to isolate phage from sewage samples as the previously described (Han et al., 2021).

### Purification of Bacteriophage BUCT603

Purification of the bacteriophage BUCT603 was performed as described previously with slight modifications (Uchiyama et al., 2011). Briefly, the phage was cultured with SMA118 in 100 ml of LB. After the complete lysis of *S. maltophilia*, the phage lysate was collected by centrifugation at 12,000 × *g* for 20 min at 4°C, and the supernatant was filtered through a 0.22 μm filter. Then, the phage suspension was further purified by discontinuous cesium chloride (CsCl) density gradient (*ρ* = 1.3, 1.5, and 1.7) and centrifuged at 30,000 × *g* for 2 h at 4°C. Finally, the banded phage particle was collected and dialyzed with PBS buffer (0.1 M, pH7.4).

### Electron Microscopy

To visualize phages, 30 μl of phage lysate was incubated with the carbon-coated copper grid for 10 min and stained with 2% uranyl acetate for 90 s and subsequently air dried. The morphology of the phages was examined with a transmission electron microscope (JEM-1200EX, Japan) at 80 kV.

### Host Range

A panel of 15 *S. maltophilia* strains that isolated from clinical samples were used for host range analysis by spot tests (Kim et al., 2020). Briefly, 500 μl of each test bacterial culture was mixed with 5 ml melt semisolid medium to pour the double-layer medium. Then, 2.5 μl serial dilutions of bacteriophage BUCT603 (10^−1^–10^−6^) were spotted on the lawn of each strain and cultured at 37°C for 16 h. After observing the plate, the tested bacteria which could form clear plaques were considered susceptible to phage infection.

### Optimal Multiplicity of Infection

The optimal multiplicity of infection (MOI) was determined as described elsewhere (Yang et al., 2020). Briefly, SMA118 culture (1 × 10^8^ CFU/ml) was mixed with serial dilutions of the phage stock at different MOI (0.01, 0.1, 1, 10, and 100), added in 5 ml LB broth and cultured at 37°C with shaking for 16 h. Then the mixture was centrifuged at 12,000 × *g* for 2 min to remove residual bacterial cells and filtered through a 0.22 μm filter. Finally, the phage titer was determined by soft agar overlay method with three parallel experiments. The one obtained the highest phage titer was regarded as the optimal MOI.

### Adsorption Assay and One-Step Growth Curve

An adsorption assay was performed according to the protocol described before (Han et al., 2021). The exponentially growing host strain SMA118 (2.0 × 10^8^ CFU/ml) was co-cultured with the phage suspensions (MOI = 0.1) at 37°C. During this period, 200 μl mixtures were, respectively, collected at 0, 2, 4, 6, 8, 10, 15, 20, 25, 30 min and centrifuged at 12,000 × *g* for 2 min. Then, 100 μl supernatant was taken to determine the number of un-adsorbed phages by the soft agar overlay method.

To further reveal the lytic cycle of bacteriophage BUCT603, one-step growth curve was performed as described previously with minor modifications (Tang et al., 2018). Briefly, liquid cultures of logarithmically growing SMA118 were infected with bacteriophage BUCT603 at a MOI of 0.1 and allowed to adsorb at 37°C for 10 min. The non-adsorbed phage was removed by centrifugation at 12,000 × *g* for 2 min, and the pellet was resuspended in 20 ml of LB broth and cultured at 37°C with shaking for up to 120 min. Samples were taken every 10 min, serially diluted, and the phage titer was determined by soft agar overlay method with three parallel experiments. One-step growth curve was drawn by taking infection time as the abscissa and phage titer as the ordinate.

### Thermal and pH Stability

To determine the thermostability of bacteriophage BUCT603, the phage suspension was incubated at various temperatures (4°C, 25°C, 37°C, 45°C, 55°C, 65°C and 75°C) for 12 h. For the pH stability analysis, the phage suspension was incubated in LB broth at different pH levels ranging from 2 to 13 for 12 h. The phage titer was determined by soft agar overlay method with three parallel experiments. The relative titer was calculated as the ratio of phage titers treated with different temperature or pH levels to those by the original level.

### Molecular Genetic Methods for Identifying Adsorbed Receptor

A random *S. maltophilia* ATCC13637 mutant pool was generated using the EZ-Tn5™<KAN-2>Tnp Transposome™ Kit (Epicentre^®^, Lucigen, United States). The electroporation of ATCC13637 competent cells were prepared as described previously (Ye et al., 2014). Then 50 μl of electrocompetent cell was mixed with 2 μl EZ-Tn5 transposome and electroporated using a Bio-Rad GenePulser Xcell™ (United States) with pulse controller set at 1.8 KV, 25 μF, 200 Ω and 0.1 cm cuvette width (Ye et al., 2014). Immediately followed by the cell revitalization, the sample was suspended in 1 ml of LB broth and incubated at 37°C for 2 h and centrifuged at 5,000 × *g* for 5 min, and discard 900 μl supernatant and resuspend the rest. Finally, bacterial suspension was mixed with 100 μl phage BUCT603 (10^9^ PFU/ml) and serially diluted with PBS (pH 7.4), and coated on LB agar plate with kanamycin for incubating at 37°C.

Surviving colonies were isolated by streaking three times on LB agar plate with kanamycin, and resistant clones were selected in a plaque assay. The genomes of the resistant clones were extracted and digested with SaIl restriction endonuclease (NEB) and ligated using T4 DNA ligase (NEB), followed amplified by PCR using primer pairs KAN-2FP-1 and KAN-2RP-1 (Supplementary Table S2). Finally, the amplification products were sent to RuiBiotech (Beijing, China) for Sanger sequencing to identify the Tn5 transposon insertion site.

Complementation of mutant clone was performed as previously described (McCutcheon et al., 2018) with some modifications. Briefly, the tonB gene was amplified from *S. maltophilia* ATCC13637 by colony PCR using primer pairs tonB-F and tonB-R (Supplementary Table S2). The resulting products were digested with HindIII and BamHI restriction endonucleases (NEB) and ligated using T4 DNA ligase (NEB) into the vector pBBR1MCS1 (Kovach et al., 1994) for expression in tonB mutant clone, ATCC13637ΔtonB.

### Extraction and Sequencing of Bacteriophage BUCT603 Genome

Phage BUCT603 genomic DNA was extracted using the phenol-chloroform method as previously reported (Han et al., 2021). Then, a DNA genomic library was constructed using NEBNext Ultra II FS DNA Library Prep Kit (NEB) as manufacturer’s instructions, and then sequenced on the Illumina NovaSeq sequencing platform with paired-end 300 nucleotide reads. The raw sequencing data containing 4,868,448 reads was analyzed using the quality control software FastQC v0.11.5 and filtered for low quality reads using Trimmomatic 0.36 with default parameters to generate 4,789,184 high-quality reads (Bolger et al., 2014). SPAdes v3.13.0 with default parameters (Bankevich et al., 2012) was used to assemble a 44,912 bp contig with 4,729,536 reads mapping to the contig with no gaps or ambiguous sites.

### Genome Annotation and Genomic Analysis

Open reading frames (ORFs) were predicted using rapid annotations of technology (RAST^1^; Overbeek et al., 2014). The protein functions were annotated using protein basic local alignment search tool (BLASTp) of NCBI server (Altschul et al., 1997).^2^ Phage genome annotation was visualized using CLC Main Workbench, version 7.7.3 (CLC Bio-QIAGEN, Aarhus, Denmark). Theoretical molecular weight of the proteins was identified using Expasy ProtParam tool (Wilkins et al., 1999).^3^ The search for putative tRNA encoding genes was performed with tRNAscan-SE v.2.0 (Lowe and Chan, 2016).^4^ The antibiotic resistance genes and virulence factors in the phage genomes were predicted by the online prediction platform ResFinder^5^ (Bortolaia et al., 2020) and VirulenceFinder^6^ (Joensen et al., 2014), respectively.

Finally, based on major capsid and DNA polymerase, phylogenetic analysis was performed with MEGA7 by the neighbor-joining method (Kumar et al., 2016). Phage homology calculations were performed using VIRIDIC (Moraru et al., 2020).^7^

### Protein Analysis of Bacteriophage BUCT603

The structural proteins of BUCT603 were predicted using the PhANNs neural networks (Cantu et al., 2020).^8^ To determine the structure proteins of BUCT603, the highly purified phage particles (10^11^ PFU/ml) were separated using SDS-PAGE method (Boulanger, 2009). Briefly, 10 μl sample with 2 μl protein loading buffer (6×) was heated at 100°C for 10 min, and was subjected by electrophoresis through 10% polyacrylamide gels run at 180 V for 50 min followed by coomassie blue staining. In addition, in order to fully understand the protein profile of BUCT603, the phage particles were sent to Beijing Huada Protein Research and Development Center for HPLC–MS analysis.

### Bacteriolytic Activity of Bacteriophage BUCT603 *in vitro*

The bacteriolytic activity of bacteriophage BUCT603 against SMA118 *in vitro* was tested as previously described with some modifications (Liu et al., 2020). Briefly, the exponential growth strains (2.0 × 10^8^ CFU/ml) were cultivated with the phage with a MOI of 0.01, 0.1, 1, and 10, followed by incubation at 37°C with moderate shaking for 16 h. *Stenotrophomonas maltophilia* cultures mixed with LB broth were used as control. In addition, in order to evaluate the effect of phage combination, phage IME15 which also could infect SMA118 was added the experimental group with MOI = 1 after cultured for 8 h. The absorbance (OD_600_) of the cultures were obtained with intervals of 1 h using NanoDrop one (Thermo, United States). The experiment was performed in triplicate.

### *Stenotrophomonas maltophilia* Infection and Phage Therapy in Mouse Model

BALB/c mice, 3–5 weeks old, weighing 15–18 g, were obtained from SPF (Beijing, China) Biotechnology Co., Ltd. and used in this study. All animal experiments followed the ARRIVE guidelines and were approved by the Ethics Committee of Chinese PLA Army General Hospital.

In order to establish the infection model of *S. maltophilia* in mouse, the effect of different concentrations of SMA118 on the survival of mice after infection was investigated. SMA118 was cultured overnight and collected by centrifugation, washed twice with phosphate buffer saline (PBS) and then the number of live cells was determined by serial dilution plate method, and finally diluted with PBS to the corresponding cell density. Mice were immunosuppressed by injected cyclophosphamide (Hu et al., 2020; CTX, 125 mg/kg) and CTX (125 mg /kg) with dexamethasone (12.5 mg/kg), respectively, for 4 days and 1 day before infection. Subsequently, the mice were divided into five groups of 10 as follows: group 1 mice were given 40 μl PBS intranasally, group 2–5 mice were given 2 × 10^6^ CFU/mouse, 4 × 10^6^ CFU/mouse, 2 × 10^7^ CFU/mouse and 3.2 × 10^7^ CFU/mouse SMA118 intranasally, respectively.

The appropriate bacterial concentrations were selected and phage therapy was performed in a similar manner as described above. Mice were immunosuppressed and divided into five groups of 10 as follows: group I mice were given 40 μl PBS and then given 2 × 10^7^ PFU/mouse BUCT603 at 6 h post-infection (hpi), group II mice were given 2 × 10^7^ CFU/mouse SMA118 and then given 40 μl PBS at 6 hpi, group III mice were given 2 × 10^7^ CFU/mouse SMA118 and then given 2 × 10^5^ PFU/mouse BUCT603 at 6 hpi, group IV mice were given 2 × 10^7^ CFU/mouse SMA118 and then given 2 × 10^6^ PFU/mouse BUCT603 at 6 hpi, group V mice were given 2 × 10^7^ CFU/mouse SMA118 and then given 2 × 10^7^ PFU/mouse BUCT603 at 6 hpi. The survival percentages were monitored in the next 168 h.

### Data Availability

**Figure 1:**
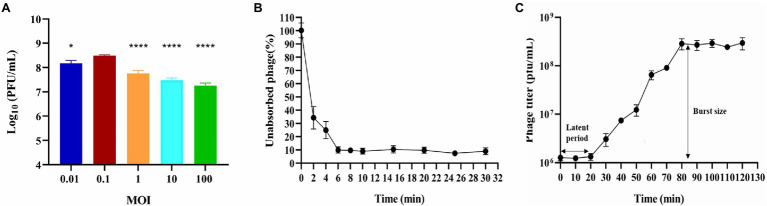
Biological characterization of phage BUCT603. **(A)** Multiplicity of infection (^***^p < 0.0001 or ^*^p < 0.05 indicates a significant difference compared to the MOI 0.1); **(B)** Adsorption rate; **(C)** One-step growth curve. Results are presented as mean values ± SD.

**Figure 2:**
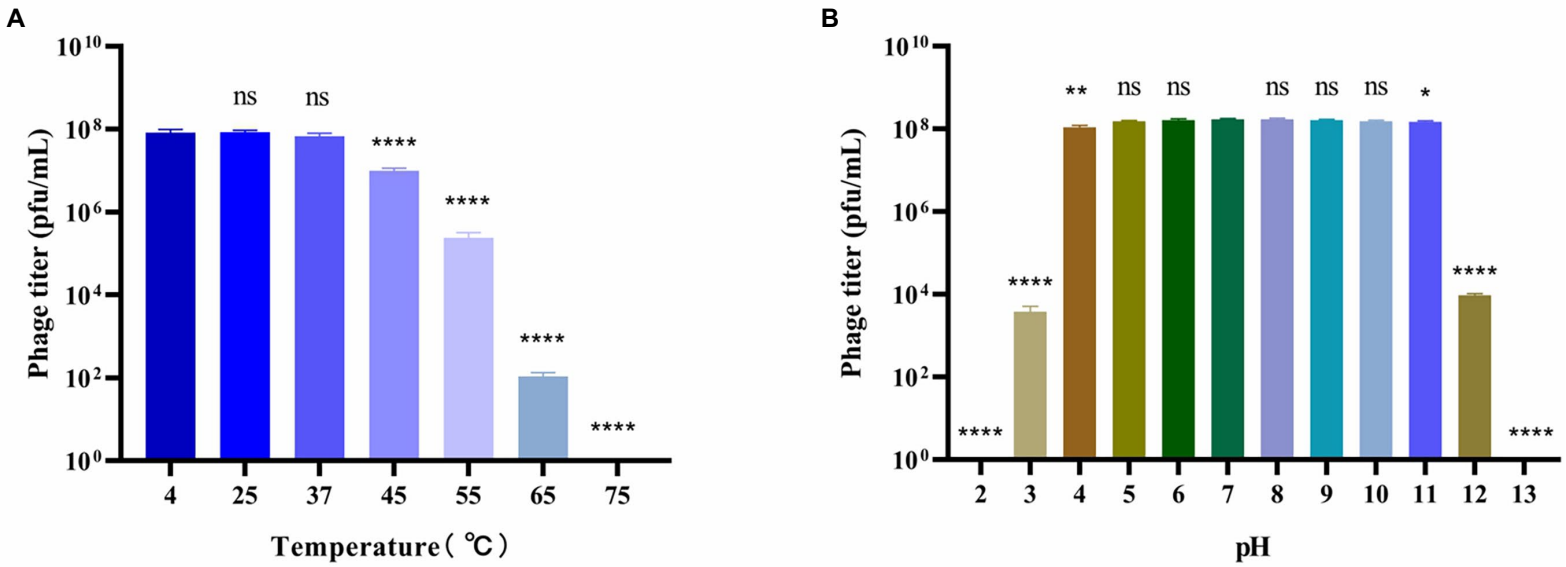
Stability of bacteriophage BUCT603. **(A)** Thermal stability of the phage treated with different temperature for 12 h; **(B)** pH stability of the phage treated with different pH for 12 h. Data are shown as the mean ± SD, ^****^p < 0.0001, ^**^p < 0.01 and ^*^p < 0.05 indicates a significant difference between this group and the control.

All data were analyzed using the GraphPad Prism 8.0.1 and expressed as means and standard deviation values. One-way analysis of variance is used in Figures 1A, 2. The complete genome sequence of bacteriophage BUCT603 has been deposited in GenBank under the accession number MW934263.

## Results and Discussion

### Isolation and Morphology of Bacteriophage BUCT603

**Figure 3:**
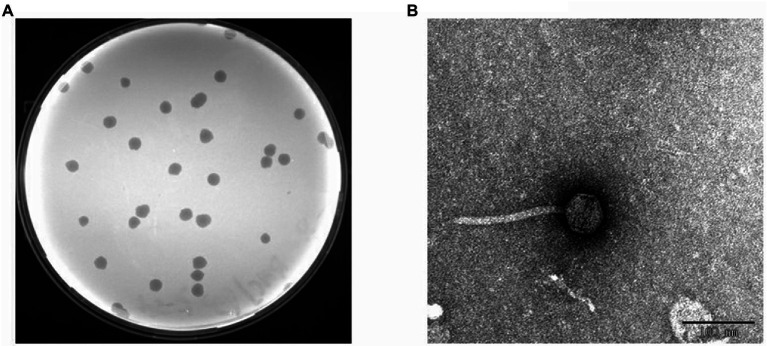
Isolated phage BUCT603. **(A)** Plaques on double agar plate. **(B)** Electron micrographs of phage BUCT603.

Several sewage samples from different hospitals were collected and tested for the presence of bacteriophages that infect clinical drug-resistant strains of *S. maltophilia*. Bacteriophage BUCT603 was isolated from sewage from the 307 Hospital of PLA using clinical SMA118. BUCT603 formed large, clear and irregular circular plaques (~3 mm in diameter) after incubation on the lawn of SMA118 for 16 h at 37°C (Figure 3A). Several other studies have also reported the presence of *S. maltophilia* lytic phages in sewage (Fan et al., 2012; Huang et al., 2012; Zhang et al., 2021) indicating that hospital sewage provides a resource for the isolation of phages directed against drug-resistant bacterium.

Transmission electron microscopy (TEM) analysis showed that BUCT603 has an icosahedral head 55.23 ± 1.45 nm in diameter (*n* = 10), a non-contractile tail 151.64 ± 2.40 nm in length (*n* = 10) and 9.0 ± 1.13 nm in width (*n* = 10; Figure 3B). No tail fibers were observed on the TEM images. Based on its morphological characteristics, bacteriophage BUCT603 belongs to the family *Siphoviridae*. Members of *Siphoviridae* are the most abundant tailed phages in public databases and have attracted an upsurge interest in their infection mechanisms among researchers (Goulet et al., 2020). The isolation and characterization of a novel *Siphoviridae* phage should provide basic data for the further study of the phage infection mechanism and the development of biological control agents.

### Host Range

The host range of a phage plays an important role in in its therapeutic applications. To estimate the potential applications of BUCT603 in phage therapy, 14 clinical drug-resistant isolates of *S. maltophilia* (Supplementary Table S1) from different hospitals and one model strain were used to test its lytic spectrum (Table 1). Phage BUCT603 infected 10 of the 15 strains tested, with relatively high lysis rates (66.7%) compared with other *S. maltophilia* lytic phages (AXL3 17.2%, DLP1 29.6%, DLP2 33.3%, and DLP6 48.1%; Peters et al., 2015, 2017; McCutcheon et al., 2020). All these data suggest that bacteriophage BUCT603 has a broad host range and a potential capacity to control *S. maltophilia* infections. Further *S. maltophilia* strains must be collected in the future to further clarify the host range of BUCT603.

**Table 1:** Host range analysis of bacteriophage BUCT603 against 15 strains.

Species	Strains	ST type	Susceptibility	Origin
*Stenotrophomonas maltophilia*	ATCC13617	N/A	+	Standard strain library
	35	ST461	++	307 hospital
	118	ST4	+++	307 hospital
	209	ST463	−	307 hospital
	532	ST296	+	307 hospital
	548	ST190	−	307 hospital
	690	ST115	+	307 hospital
	824	ST413	−	307 hospital
	826	ST378	+	307 hospital
	992	ST8	+	307 hospital
	1,207	ST502	+	307 hospital
	1,209	ST7	−	210 hospital
	1,284	ST111	+++	210 hospital
	1,785	ST31	++	210 hospital
	1,786	ST362	−	210 hospital

+, plaques at 10^−1^–10^−2^; ++, plaques at 10^−3^–10^−4^; +++, plaques at 10^−5^–10^−6^; −, no sensitivity to phage.

### Optimal MOI

Multiplicity of infection refers to the ratio of bacteriophage to bacteria at the time of infection, which can provide a reference for the therapeutic applications of the bacteriophage. Bacteriophage BUCT603 was mixed with the host SMA118 at different MOIs. When the MOI was 0.1, BUCT603 produced the maximum titer of virions (Figure 1A), indicating that 0.1 is the optimal MOI of BUCT603.

### Adsorption Assay and One-Step Growth Curve

The adsorption of BUCT603 to cells of its host SMA118 was very efficient. Over 90% of the phages had effectively adsorbed after incubation for 6 min (Figure 1B), which differed from the adsorption rates of the previously characterized *S. maltophilia* phages Smp14 and phiSMA5, which were 85 and 70% after 5 and 50 min, respectively (Chang et al., 2005; Chen et al., 2007).

One-step growth curve can quantitatively describe the characteristic growth of a phage and visualize its latent period and burst size. The one-step growth curve of bacteriophage BUCT603 was analyzed by infecting exponentially growing strains with the phage at an MOI of 0.1. As shown in Figure 1C, the latent period of bacteriophage BUCT603 was ~20 min. There was then a gradual increase in the number of viral particles released, and BUCT603 entered the plateau period at 80 min. The burst size of bacteriophage BUCT603, defined as the phage titer in the plateau phase divided by the mean phage titer in the latent phase (Lee et al., 2019), was about 233 ± 61 pfu per bacterial cell. Other *S. maltophilia* lytic phages that have been characterized, Smp14, phiSMA5 and IME15, have latent periods of 20, 80, and 30 min, respectively, and the average burst size was 150, 95, and 158 pfu per bacterial cell, respectively (Chang et al., 2005; Chen et al., 2007; Huang et al., 2012). Compared with these three phages, bacteriophage BUCT603 showed a relatively short latent period and a large burst size, explaining its strong lytic activity against *S. maltophilia*.

### Stability of Bacteriophage BUCT603

To better understand the biological characteristics of bacteriophage BUCT603, we studied the influence of temperature and pH on its stability. The thermal stability of phage BUCT603 is shown in Figure 2A. No significant reduction in phage viability was observed under a range of temperatures (4°C–37°C). However, the phage titer began to decrease significantly when the phage was incubated at 45°C for 12 h, and even no phage survived at 75°C, indicating that BUCT603 is sensitive to temperature and not suitable for apply in high temperature environment. The pH stability of phage BUCT603 was measured in a broad range of pH values from 2 to 13. BUCT603 was most stable in the pH range of 5–10 (Figure 2B). However, BUCT603 was deactivate under highly acidic conditions (pH < 3) and highly alkaline conditions (pH > 12). Since BUCT603 is sensitive to temperature, the storage temperature should be paid attention in future therapeutic applications.

### Adsorption Receptor Identification

**Figure 4:**
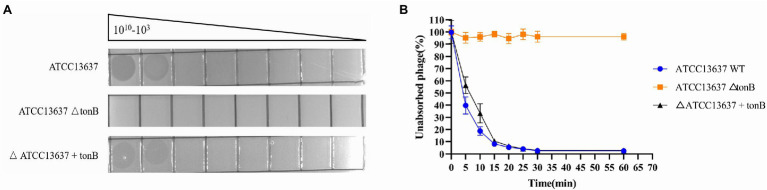
Bacteriophage BUCT603 uses tonB protein as an adsorption receptor to infect *Stenotrophomonas maltophilia* ATCC13637. **(A)** Wildtype *S. maltophilia* ATCC13637 is susceptible to BUCT603 infection, whereas the ΔtonB mutant strain is resistant to phage infection, but complementation of ATCC13637 ΔtonB with the ATCC13637 tonB gene restores phage infection to wildtype levels. **(B)** BUCT603 can rapidly adsorb wildtype *S. maltophilia* ATCC13637, whereas BUCT603 cannot adsorb ΔtonB mutant strain, but BUCT603 restores adsorption to complementation of ATCC13637 ΔtonB with the ATCC13637 tonB gene.

To identify potential receptors for bacteriophage BUCT603 infection, we used *S. maltophilia* ATCC13637 (GenBank accession number: CP008838.1), which has a clear gene sequence, as the subject of our study. We constructed the transposon insertion random mutagenesis library of ATCC13637 by using Tn5 transposon, and through plaque screening we obtained the resistant clones of BUCT603. By sequencing the Tn5 insertion site sequences of the four resistant clones, we found that the transposons were inserted in the gene encoding TonB-dependent receptor family protein (Protein ID: AIL08586.1). We performed plaque and adsorption assays on the resistant clones and found that BUCT603 no longer adsorbed and lysed ATCC13637ΔtonB, whereas the complementary strains with the resistance mutation regained the sensitivity to BUCT603 (Figure 4). On the basis of similar findings in other phages (Killmann and Braun, 1994; Rabsch et al., 2007), that TonB protein is suggested as the receptor for BUCT603 infection of ATCC13637. Among the *S. maltophilia* phages characterized to date, AXL3, DLP1, DLP2, DLP3, and DLP4 (Peters et al., 2015, 2019, 2020; McCutcheon et al., 2020) use the type IV pilus as their receptor, and it is the only reported receptor for *S. maltophilia* phages. In this study, the adsorption receptor of BUCT603 was identified as TonB, which extends the known phage receptors of *S. maltophilia*. Whether the infection of other hosts by BUCT603 is dependent on TonB requires further investigation.

TonB systems of Gram-negative bacteria play an important role in transportation of nutriment from outside the cell. TonB systems consist of plasma membrane proteins ExbB-ExbD and periplasmic protein TonB, and provide the energy required by TonB-dependent receptors to transport substrates (Schauer et al., 2008). Because the TonB protein is associated with bacterial virulence (Wang et al., 2008; Porcheron et al., 2013), if a bacterium becomes resistant to a phage by modifying its TonB protein, this resistance will potentially reduce the virulence and fitness of the bacterium relative to those of non-resistant bacteria. Therefore, we believe that BUCT603, which uses TonB as a receptor, will be a promising candidate for the treatment of *S. maltophilia* infection.

### Analysis of Bacteriophage BUCT603 Genome

The whole genome of phage BUCT603 is composed of 44,912 bp of linear double-stranded DNA with a G + C content of 63.7%. In a Basic Local Alignment Search Tool (BLASTn) analysis, the coverage of the BUCT603 genome compared with other phages is only 1%, indicating that BUCT603 is a novel phage infecting *S. maltophilia* and quite different from other known bacteriophages. The complete annotations of the BUCT603 genome with supporting evidence are provided in Table 2. Based on RAST and BLASTp analyses, the BUCT603 genome was predicted to encode 64 ORFs, but only 19 ORFs (30%) had significant homology to proteins with known functions. No tRNAs genes were identified in the BUCT603 genome with the tRNAscan-SE software. Our failure to identify either virulence or antibiotic resistance genes in bacteriophage BUCT603 indicates that it is safe for therapeutic use at the genomic level.

**Table 2:** Genome annotation of bacteriophage BUCT603.

Gene	Start	Stop	Strand	Length (AA)	Putative function	Best-match BLASTp result	Query cover (%)	E-values	Identity (%)	Accession	MW (kDa)
gp01	571	227	−	114	HNH endonuclease	Xanthomonas phage phiL7	100	2e-41	59.48	YP_009998229.1	13.2
gp02	1,009	1,362	+	117	Hypothetical protein	Not hit	–	–	–	–	12.7
gp03	1,428	1,712	+	94	Hypothetical protein	Not hit	–	–	–	–	10.2
gp04	1,784	2,161	+	125	Hypothetical protein	Not hit	–	–	–	–	14.2
gp05	2,226	2,444	+	72	Hypothetical protein	Not hit	–	–	–	–	8.1
gp06	2,458	2,688	+	76	Hypothetical protein	Not hit	–	–	–	–	8.6
gp07	2,748	3,338	+	196	Hypothetical protein	Not hit	–	–	–	–	21.3
gp08	3,335	3,592	+	85	Hypothetical protein	Not hit	–	–	–	–	9.4
gp09	3,589	3,810	+	73	Hypothetical protein	Not hit	–	–	–	–	8.3
gp10	3,803	4,054	+	83	Hypothetical protein	Not hit	–	–	–	–	9.5
gp11	4,116	4,271	+	51	Hypothetical protein	Not hit	–	–	–	–	5.9
gp12	4,268	4,444	+	58	Hypothetical protein	Not hit	–	–	–	–	6.8
gp13	4,434	4,892	+	152	Hypothetical protein	Not hit	–	–	–	–	15.9
gp14	4,889	5,065	+	58	Hypothetical protein	Not hit	–	–	–	–	6.5
gp15	5,137	5,262	+	41	Hypothetical protein	Not hit	–	–	–	–	4.9
gp16	5,316	5,555	+	79	Hypothetical protein	Not hit	–	–	–	–	8.6
gp17	5,559	5,732	+	57	Hypothetical protein	Not hit	–	–	–	–	6.9
gp18	5,886	6,485	+	199	Hypothetical protein	Not hit	–	–	–	–	21.8
gp19	6,482	6,787	+	101	Hypothetical protein	Not hit	–	–	–	–	11.7
gp20	6,857	7,132	+	91	Hypothetical protein	*Xanthomonas translucens*	65	2e-04	42.86	WP_058358323.1	10.0
gp21	7,129	7,425	+	98	Hypothetical protein	*X. translucens*	100	8e-18	43.43	WP_058358322.1	11.3
gp22	7,438	7,770	+	110	Hypothetical protein	Not hit	–	–	–	–	11.9
gp23	7,770	7,895	+	41	Hypothetical protein	Not hit	–	–	–	–	4.5
gp24	8,024	8,845	+	273	DNA primase	Xanthomonas phage phiL7	95	2e-113	60.46	YP_002922662.1	31.0
gp25	8,842	10,143	+	433	DNA helicase	Xanthomonas phage phiL7	99	2e-172	54.63	YP_002922661.1	49.0
gp26	10,186	10,740	+	184	Hypothetical protein	Bacteriophage Titan-X	94	8e-19	33.33	QGH45048.1	20.2
gp27	10,742	13,246	+	834	DNA polymerase A	Xylella phage Prado	99	0.0	62.98%	YP_008859402.1	93.4
gp28	13,259	14,098	+	279	hypothetical protein	*X. translucens*	97	2e-91	52.38	WP_058358272.1	31.2
gp29	14,167	15,105	+	312	5′-3′ exonuclease	Xanthomonas phage phiL7	99	3e-95	51.27	YP_002922655.1	34.6
gp30	15,102	15,506	+	134	DNA endonuclease VII	Xylella phage Prado	91	6e-46	57.72	YP_008859405.1	14.5
gp31	15,519	16,418	+	299	Ribonuclease H-like domain-containing protein	*X. translucens*	97	2e-157	69.52	WP_081048563.1	34.4
gp32	16,532	17,359	+	275	Hypothetical protein	Xanthomonas phage phiL7	49	5e-27	49.28	YP_002922651.1	30.2
gp33	17,435	18,268	+	277	Hypothetical protein	Not hit	–	–	–	–	29.8
gp34	18,274	18,414	+	46	Hypothetical protein	Not hit	–	–	–	–	4.97
gp35	18,405	18,809	+	134	Hypothetical protein	Not hit	–	–	–	–	14.5
gp36	18,814	21,279	+	821	T3/T7-like RNA polymerase	Xanthomonas phage phiL7	99	0.0	55.42	YP_002922649.1	93.5
gp37	21,291	21,665	+	124	Hypothetical protein	*Stenotrophomonas maltophilia*	63	5e-24	62.20		13.1
gp38	21,736	21,951	+	71	Hypothetical protein	Not hit	–	–	–	–	8.3
gp39	21,948	22,154	+	68	Hypothetical protein	Not hit	–	–	–	–	7.1
gp40	22,151	22,846	+	231	Hypothetical protein	Not hit	–	–	–	–	26.4
gp41	22,896	23,102	+	68	Hypothetical protein	Not hit	–	–	–	–	7.6
gp42	23,384	23,148	−	78	o-spanin	Xylella phage Prado	94	2e-24	58.11	YP_008859436.1	8.75
gp43	24,160	23,633	−	175	Lysozyme	Xanthomonas phage phiL7	89	3e-78	67.31	YP_002922642.1	19.1
gp44	24,414	24,881	+	155	Hypothetical protein	*S. maltophilia*	87	1e-10	28.80	VUM05985.1	17.4
gp45	25,497	24,868	−	209	Hypothetical protein	*S. maltophilia*	100	2e-94	66.19	WP_099473297.1	22.9
gp46	25,832	25,536	−	98	Hypothetical protein	*S. maltophilia*	100	3e-43	72.45	WP_164186364.1	10.8
gp47	27,020	25,836	−	394	Hypothetical protein	phage BUCT555	30	3e-06	31.15	QQM14856.1	42.2
gp48	31,805	27,063	−	1,580	Putative tail component protein	Xanthomonas virus OP1	99	0.0	41.30	YP_453579.1	172.1
gp49	32,173	31,793	−	126	Peptidoglycan endopeptidase	Stenotrophomonas sp.	92	3e-26	47.11	MTI72244.1	14.8
gp50	32,591	32,154	−	145	Hypothetical protein	*X. translucens*	99	9e-68	68.06	WP_058358293.1	16.0
gp51	32,973	32,605	−	122	Hypothetical protein	*X. translucens*	95	4e-46	56.90	WP_058358292.1	13.8
gp52	36,301	32,990	−	1,103	Tail tape measure protein	Xanthomonas phage phiL7	88	0.0	43.63	YP_002922630.1	117.8
gp53	36,421	36,305	−	38	Hypothetical protein	*Xanthomonas citri* pv. citri	97	1e-06	59.46	MBD4920667.1	4.3
gp54	36,915	36,613	−	100	Hypothetical protein	Xanthomonas virus OP1	100	8e-27	46.00	YP_453571.1	11.2
gp55	37,574	36,915	−	219	Phage tail protein	*X. translucens*	99	4e-99	66.36	WP_058358297.1	23.5
gp56	37,993	37,601	−	130	hypothetical protein	*X. translucens*	63	1e-20	45.78	WP_058358296.1	14.7
gp57	38,459	37,995	−	154	Hypothetical protein	*X. translucens*	91	3e-41	51.47	WP_196480298.1	17.0
gp58	38,820	38,443		125	Hypothetical protein	Xanthomonas virus CP1	94	0.005	29.17	YP_007238081.1	13.8
gp59	39,253	38,822	−	143	Phage gp6-like head-tail connector protein	*X. translucens*	85	5e-17	38.52	WP_058358294.1	15.9
gp60	40,511	39,309	−	400	Major capsid protein	Xanthomonas phage phiL7	95	1e-173	64.04	YP_002922620.1	43.0
gp61	41,326	40,541	−	261	Clp protease ClpP	*X. translucens*	91	2e-90	60.42	WP_058358269.1	27.7
gp62	42,667	41,327	−	446	Phage portal protein	*X. translucens*	98	3e-162	56.69	WP_081048562.1	48.9
gp63	44,413	42,680	−	577	Terminase large subunit	Xanthomonas sp. 3,498	96	0.0	57.96	YP_002922617.1	63.6
gp64	44,781	44,410	−	123	Hypothetical protein	Mizugakiibacter sediminis	80	2e-14	50.00	WP_062537393.1	13.2

**Figure 5:**
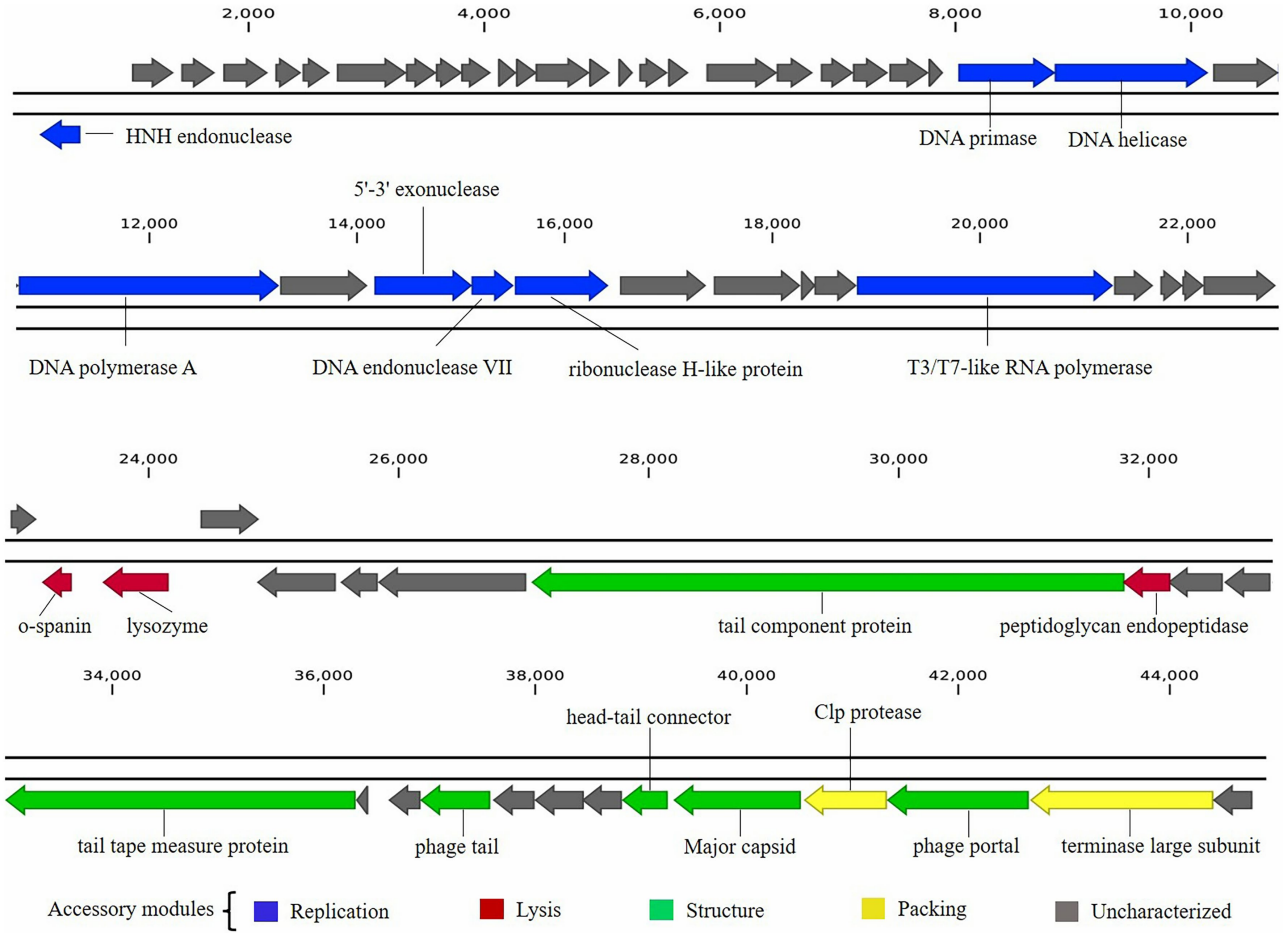
Graphical representation of phage BUCT603 genome. Colors indicate the functional categories of the genes, and arrows indicate the orientation of each ORF.

The functional proteins encoded in the phage BUCT603 genome can be classified into at least four categories: packaging proteins, structural proteins, DNA replication proteins, and lysis-related proteins, and are visualized in Figure 5.

Five proteins encoded by phage BUCT603 were predicted to be involved in DNA packaging: HNH endonuclease protein (gp01), DNA endonuclease VII protein (gp30), Clp protease protein (gp61), and terminase large subunit (gp63). The gp01-encoded protein shares 59% identity with HNH endonuclease, which has been identified in the *Xanthomonas* phage phiL7 (GenBank accession number: NC_012742.1). HNH endonucleases are present in many bacteriophages and play important roles in the DNA packaging machinery. The gp30-encoded protein is similar (58% identity) to DNA endonuclease VII from the Xylella phage Prado (GenBank accession number: NC_022987.1). DNA endonuclease VII is involved in completion of packaging (Kala et al., 2014). The terminase of bacteriophage, a hetero-oligomer composed of a large subunit and a small subunit, is mainly involved in the translocation of DNA inside the capsid (Feiss and Rao, 2012). The protein encoded by gp63 is similar (58% identity) to the terminase large subunit of phage phiL7. Although the terminase small subunit is not annotated in the BUCT603 genome using BLASTp analyses, we speculate that the gp64 in this co-directionally oriented gene cluster probably encodes the terminase small subunit. Gp61 is predicted to encode the Clp protease protein, which is essential for procapsid production. In double-stranded DNA phages, the procapsid matures and packages the DNA (Liu and Mushegian, 2004). Phage gene gp62, encoding the portal protein, is thought to regulate packaging termination through DNA-dependent conformational changes during packaging. The portal protein also plays an important role in DNA entry and exit from the viral capsid at the beginning of infection (Isidro et al., 2004).

Six ORFs in the phage BUCT603 genome are predicted to encode phage structural proteins, and these genes are scattered on the negative strand of the genome (Figure 5). These predicted proteins function in the capsid structure (gp60 and gp62), tail structure (gp48, gp52, and gp55) and connection structure (gp59). Gp48 and gp55 are predicted to encode a putative tail component protein and a phage tail protein, respectively. Phage tail proteins mainly mediate the adsorption of phages to their hosts and their penetration of the outer membrane of host cells after infection. Therefore, their structure largely determines the host range of the phage (North et al., 2019). Gp52 is predicted to encode a tail tape-measure protein, which dictates the tail length and facilitates the transit of the DNA to the bacterial cell cytoplasm during infection (Belcaid et al., 2011). The head-tail connector protein, predicted to be encoded by gp59, acts as an adaptor and directly binds to portal proteins during connector assembly (Tavares et al., 2012). A BLASTp analysis of the gp60-encode protein shows high similarity (64% identity) to the major capsid protein of phage phiL7. The phage capsid can protect the phage genome from the harsh extracellular environment, and the major capsid gene of phage as one of the conserved virion genes is also used for phage classification (Tétart et al., 2001).

A protein comparison showed that the phage BUCT603 genome encodes six proteins related to DNA replication and repair, including DNA primase (gp24), DNA helicase (gp25), DNA polymerase (gp27), 5′–3′ exonuclease (gp29), ribonuclease H-like domain-containing protein (gp31), and T3/T7-like RNA polymerase (gp36). Among these proteins, those encoded by gp24, gp25, gp29, and gp36 are 60, 55, 51, and 55% identical, respectively, to the corresponding proteins encoded by phage phiL7, a T7-like phage. However, BUCT603 gp36 is located at the right end of the DNA metabolic gene cluster, a distinguishing characteristic of all reported phiKMV-like phages (Ahern et al., 2014). Moreover, the BUCT603 gp27-encoded protein shares 63% identity with the DNA polymerase of the Prado, a phiKMV-like phage. We speculate that the DNA replication mechanisms of phage BUCT603 is analogous to that of phiKMV-like phages.

Three proteins related to the release of the phage at the end of the latent period were predicted, namely o-spanin (gp42), lysozyme (gp43) and peptidoglycan endopeptidase (gp49). For Caudovirales of Gram-negative hosts, there are three lysis systems: the holin-endolysin, pinholin-SAR endolysin and spanins pathways (Cahill and Young, 2019). Spanins are newly discovered phage lysis proteins that are composed of an outer membrane lipoprotein (o-spanin) and an integral inner membrane protein (i-spanin). In the genome of BUCT603, gp42 encodes a spanin-like protein, shares 58% homology with o-spanin of the phage Prado, but no i-spanin-encoding gene was detected. One spanin protein may perform two roles in BUCT603, as in the T1 phage (Summer et al., 2007). The protein encoded by gp43 shares 67% identity with the lysozyme of phage Prado, and plays a role in the release of virions by lysing the host cell or in facilitating infection, as do the lysozymes of other phages (Trudil, 2015). Interestingly, gp49 is predicted to encode a peptidoglycan endopeptidase, which plays a role in the degradation of cell wall and has been found in the phages of Gram-positive bacteria (Loessner et al., 1995; Reynolds et al., 2001). Further studies are required to clarify the lysis mechanism of BUCT603.

### Phylogenetic Analysis of Bacteriophage BUCT603

**Figure 6:**
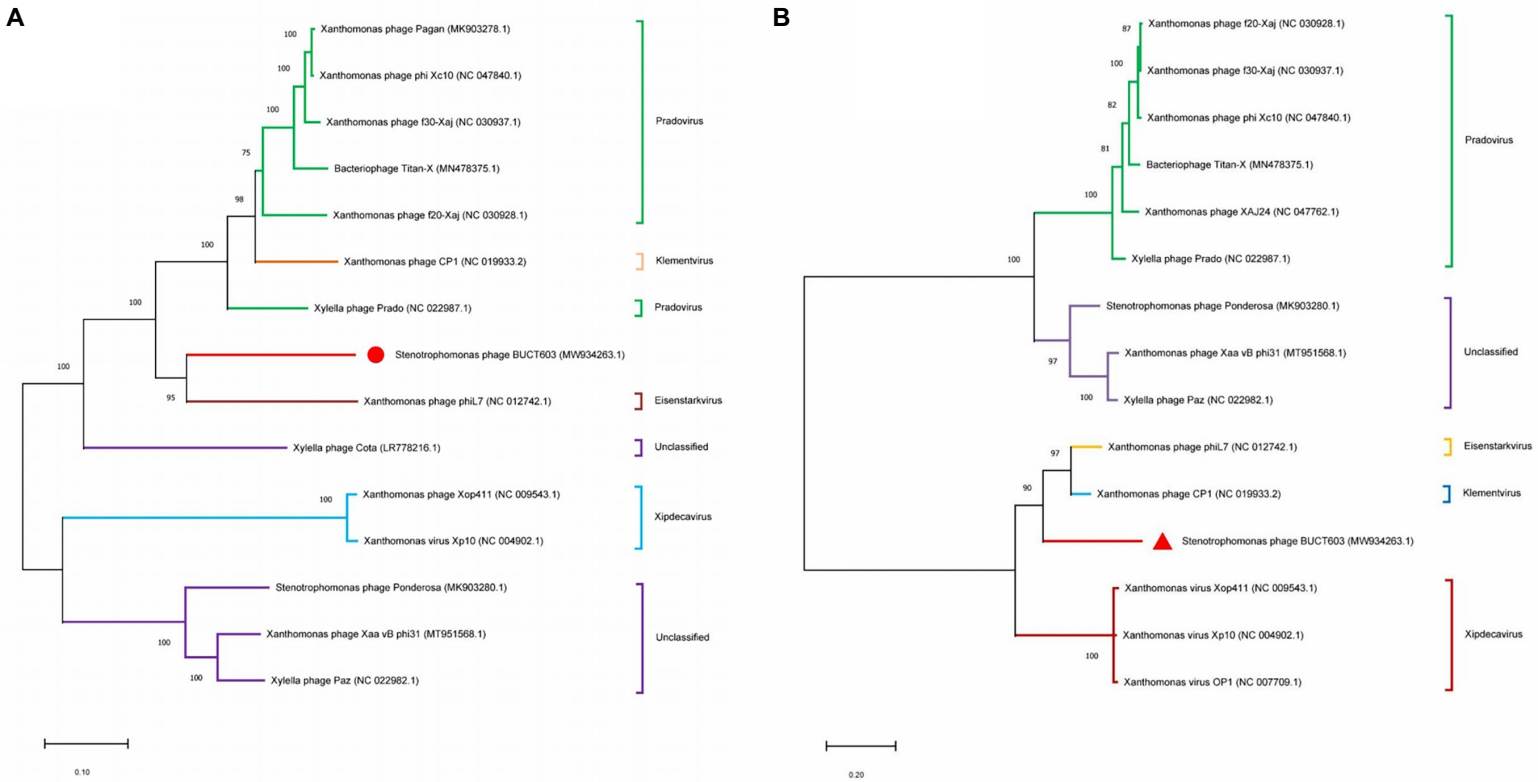
Phylogenetics tree were formed by DNA polymerase **(A)** and major capsid **(B)** of phage. The amino acid sequences of the related phages were downloaded from NCBI. The phylogenetic tree was checked by Bootstrap method, and the number of test replicates is 1,000 times.

To clarify the evolutionary relationships between bacteriophage BUCT603 and similar phages, the DNA polymerase (gp27) and terminase large subunit (gp63) proteins were used to construct a phylogenetic tree with other homologous proteins obtained from the NCBI database. The phylogenetic tree of DNA polymerase protein (Figure 6A) showed that BUCT603 was on the same branch as phage phiL7, indicating the evolutionary relatives were relatively close between two phages. The phylogenetic tree of terminase large subunit protein (Figure 6B) showed BUCT603 was on the new branch. A global genome comparison map (Supplementary Figure S1) of BUCT603 and phiL7 showed that they are distinct at the protein level, and that the protein homology coverage is <30%. In addition, we used the VIRIDIC to calculate the intergenomic similarities of BUCT603 and other known phages, the result showed the maximum similarity between BUCT603 and other known sequences is 18% (Supplementary Figure S2), sufficient to qualify it as a new species. Therefore, bacteriophage BUCT603 should be grouped a new genus in the family *Siphoviridae*.

### Proteomic Analysis

The structural proteins of phage BUCT603 were analyzed with SDS-PAGE. Eight major protein bands and 10 minor protein bands were detected, with molecular masses ranging from 16 to 180 kDa (Supplementary Figure S3). To identify as many structural proteins as possible, whole phage BUCT603 particles were directly digested with trypsin, and the component detected with mass spectrometry. Using the predicted proteins of BUCT603 as the alignment database, 24 proteins were identified, representing 37.5% of the total annotated proteins (Table 3). The scores of the proteins encoded by gp60, gp48, gp55, gp62, gp31, gp59, and gp52 were >1,000, indicating that the confidence with which the proteins were predicted was high and that the similarity of their genes to genes encoding structural proteins in other characteristic phages was high. Therefore, we speculate that the gp60, gp48, gp55, gp62, gp31, gp59, and gp52 of phage BUCT603 can be categorized as structural proteins or proteins involved in the morphogenesis of the phage. The annotated lysozyme encoded by gp43, which plays a role in the release of virions by lysing the host cell (Trudil, 2015), was isolated with mass spectrometry. The remaining proteins, listed in Table 3, could not be matched to proteins of known functions in the NCBI database, but some of them were predicted to be structural proteins using PhANNs, such as gp45 and gp56 encoding proteins were assigned to minor tail class, gp50 and gp58 encoding proteins were assigned to head-tail joining class, gp45 and gp56 encoding proteins were assigned to minor tail class, gp47 encoding protein was assigned to baseplate class. In addition to the identified proteins in Table 3, gp13 and gp14 encoding proteins were assigned to portal class. Prediction and classification of bacteriophage structural proteins would be highly advantageous for identifying functional roles of proteins of bacteriophage origins. The accuracy of predicted structural proteins needs to be verified in the further research.

**Table 3:** Genes encoding virion proteins in BUCT603 identified by mass spectrometry.

No.	Predicted function	Gene no.	Mol.Mass (kDa)	No. of peptides	Coverage (%)	Protein score
1	Major capsid protein	gp60	43.04	51	40	6,516
2	Putative tail component protein	gp48	172.42	59	35	3,451
3	Phage tail protein	gp55	23.55	31	48	3,029
4	Phage portal protein	gp62	49.01	34	51	2,989
5	Ribonuclease H-like domain-containing protein	gp31	34.71	28	48	1,903
6	Phage gp6-like head-tail connector protein	gp59	15.93	17	83	1,432
7	Tail tape measure protein	gp52	117.73	46	35	992
8	Hypothetical protein	gp58	13.88	11	83	875
9	Hypothetical protein	gp56	14.72	7	62	435
10	Hypothetical protein	gp47	42.38	6	16	380
11	Hypothetical protein	gp51	13.83	11	51	348
12	Hypothetical protein	gp50	16.03	8	49	344
13	Hypothetical protein	gp45	23.07	5	29	311
14	Hypothetical protein	gp33	30.02	8	40	292
15	Hypothetical protein	gp19	11.86	5	23	55
16	Hypothetical protein	gp57	16.96	2	25	52
17	Hypothetical protein	gp44	17.63	1	11	50
18	Lysozyme	gp43	19.28	3	10	50
19	Hypothetical protein	gp18	21.92	2	14	49
20	Hypothetical protein	gp17	6.91	1	26	29
21	Hypothetical protein	gp32	30.39	1	3	27
22	Hypothetical protein	gp54	11.27	2	21	23
23	Hypothetical protein	gp07	21.47	2	11	20
24	Hypothetical protein	gp53	4.27	1	11	14

### Bacteriolytic Activity *in vitro*

**Figure 7:**
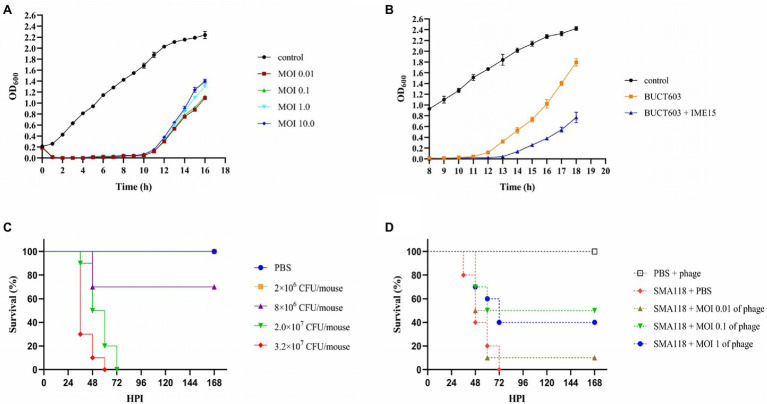
The inhibitory effect of phage BUCT603 on SMA118 *in vitro* and *in vivo*. **(A)** Growth curves of SMA118 infected with BUCT603 at different MOIs; **(B)** BUCT603 and IME15 were combined in a 1:1 ratio at MOI 0.1 to infect SMA118; **(C)** Survival curves of mice after inoculation with different concentrations of SMA118; **(D)** Survival curves of mice inoculated with SMA118 or PBS and treated with phages with different MOI.

The characterization of phage lytic activities *in vitro* is an important first step in evaluating the effect of one or more phages in treating bacterial infection. The lytic activity of phage BUCT603 was determined at different MOIs for 16 h, and uninfected SMA118 was examined in parallel as the control (Figure 7A). The absorbance of phage-BUCT603-infected bacteria at MOIs of 0.01, 0.1, 1, and 10 was significantly reduced at 1 h post-infection (hpi) and remained low at 10 hpi relative to that of the control. BUCT603 showed strong lytic activity and efficiently inhibited the growth of the host bacterium in the exponential phase. However, host lysis became inefficient after 12 h in culture, which suggests that phage-resistant strains may have emerged. This is a common phenomenon in the arms race between bacteria and phages (Hampton et al., 2020). The combination of phages IME15 and BUCT603 delayed the emergence of resistant SMA118 strains by 2 h compared with their emergence when BUCT603 was used alone, and the growth of the resistant strains that emerged was also slowed (Figure 7B). Therefore, combining different phages into a therapeutic cocktail may enhance the clinical efficacy of phage therapies.

### Efficiency of Phage Treatment *in vivo*

The effectiveness of phage BUCT603 was evaluated *in vivo* in a mouse model of *S. maltophilia* infection. To evaluate the virulence of SMA118, the survival rates of the mice were observed after infection with different bacterial concentrations. Compared with the mice in the PBS-injected group, the mice inoculated with SMA118 showed thinning hair and reduced activity. SMA118 at a concentration of 2 × 10^7^ CFU/mouse caused significant mortality at 48 h (Figure 7C). Most of the mice showed symptoms of infection at around 36 h, and higher concentrations (2 × 10^7^ or 3.2 × 10^7^ CFU/mouse) caused higher mortality rates than the lower concentrations (2 × 10^6^ or 8 × 10^6^ CFU/mouse). To evaluate the potential efficacy of bacteriophage BUCT603 against *S. maltophilia* in the clinical context, a dose of 2 × 10^7^ CFU/mouse was selected as the inoculum for the therapeutic experiment, because this dose induced a mortality rate of 100% in mice within 3 days.

In the treatment experiments, the survival rate of the uninfected group of mice (PBS + phage) was 100%, indicating that the phage was safe in this model. Compared with the positive control (SMA118 + PBS), the phage-treated groups (SMA118 + MOI 0.1 of phage or SMA118 + MOI 1 of phage) effectively reduced the mortality of the mice, with survival rates of 40 and 50%, respectively, at 168 hpi (Figure 7D). However, the phage-treated infected mice did not achieve a survival rate of 90%–100%, probably because the bacteria developed rapidly in the mice and developed resistance to the phage. It is a common issue that pathogens susceptibly develop resistance to a single-phage treatment, while combining different phages into a phage cocktail are often used in efforts to mitigate issues of phage resistance development (Li et al., 2021, 2022). Further phage cocktail treatment studies may improve the utility of BUCT603 as a potential biological agent for the treatment of drug-resistant *S. maltophilia*. To our knowledge, no other study has used an *S. maltophilia*-directed phage to treat infection in a mouse model, so this study provides an important reference for phage treatments of *S. maltophilia* infections in the future.

## Conclusion

The abuse of antibiotics in the clinical context leads to drug resistance among bacterial pathogens, including *S. maltophagia*. The horizontal or vertical transfer of drug-resistance genes leads to the gradual proliferation of drug resistance among these pathogens, increasing the difficulty of their prevention and control (Lerminiaux and Cameron, 2019). Bacteriophages are the most abundant organisms, with great genetic diversity, and are widely distributed in all ecological environments in nature. Their specificity means that bacteriophages have relatively narrow antimicrobial spectra and can only specifically inhibit a single species, or even in some cases, can only lyse certain strains within a species (Kaur et al., 2021). Therefore, a large number of bacteriophages must be isolated and studied to meet the need for bacteriophages in the prevention and treatment of clinical pathogenic bacterial infections. Phage genomic research will improve our understanding of the diversity of phage, and improve the reliability and safety of phage therapies. In this study, we characterized a *S. maltophagia* phage, BUCT603, which is highly virulent. Its genome lacks any known genes encoding toxins or other proteins potentially deleterious to humans. BUCT603 infected >60% of the *S. maltophagia* isolates tested, rapidly adsorbed to the targeted *S. maltophagia* strains, and had a relatively large burst size, indicating its ability to control and kill these bacteria rapidly and effectively. Although BUCT603 rapidly inhibited the host’s growth *in vitro* and improved the survival in infected mice, the development of resistant bacteria reduced its therapeutic efficacy. Some therapeutic results cannot be obtained with single-phage therapy, this does not mean that a single-phage therapy is not successful, but strategies must be developed to improve their effects. Combining phages with different host ranges to form phage cocktails can potentially stop bacteria from evolving resistance to these phages, and has shown promising therapeutic effects (Onsea et al., 2019; Petrovic Fabijan et al., 2020; Leitner et al., 2021). Therefore, in subsequent studies, we will evaluate the efficacy of a cocktail of phages with different receptor types for the treatment of *S. maltophagia* infections. We believe that BUCT603 could be a suitable candidate for further development as an antibacterial agent or phage therapy.

## Data Availability Statement

The datasets presented in this study can be found in online repositories. The names of the repository/repositories and accession number(s) can be found at: Genbank, MW934263.

## Ethics Statement

The animal study was reviewed and approved by Ethics Committee of Chinese PLA Army General Hospital.

## Author Contributions

PH drafted the manuscript and performed the experiments. WZ isolated the phage. MP and YL sequenced and assembled the phage genome. LS analyzed the data. XA, ML, and FL directed the experiment. SZ completed mouse experiments. HF and YT designed the research and revised the manuscript. All authors contributed to the article and approved the submitted version.

## Funding

This research was supported by Funds for First-class Discipline Construction (nos. XK1805 and XK1803-06), National Key Research and Development Program of China (nos. 2018YFA0903000, 2020YFC2005405, 2020YFA0712100, 2020YFC0840805, 19SWAQ06, and 20SWAQK22), Inner Mongolia Key Research and Development Program (no. 2019ZD006), NSFC-MFST project (China-Mongolia; no. 31961143024), and Fundamental Research Funds for Central Universities (nos. BUCTRC201917 and BUCTZY2022).

## Conflict of Interest

The authors declare that the research was conducted in the absence of any commercial or financial relationships that could be construed as a potential conflict of interest.

## Publisher’s Note

All claims expressed in this article are solely those of the authors and do not necessarily represent those of their affiliated organizations, or those of the publisher, the editors and the reviewers. Any product that may be evaluated in this article, or claim that may be made by its manufacturer, is not guaranteed or endorsed by the publisher.

## Footnotes

^1^ https://rast.nmpdr.org/

^2^ https://blast.ncbi.nlm.nih.gov/Blast.cgi

^3^ https://web.expasy.org/protparam/

^4^ http://lowelab.ucsc.edu/cgi-bin/tRNAscan-SE2.cgi

^5^ https://cge.cbs.dtu.dk/services/ResFinder/

^6^ https://cge.cbs.dtu.dk/services/VirulenceFinder/

^7^ http://rhea.icbm.uni-oldenburg.de/VIRIDIC/

^8^ https://phanns.com
