# Supplementary material for: Characterization of the Bacteriophage BUCT603 and Therapeutic Potential Evaluation Against Drug-Resistant Stenotrophomonas maltophilia in a Mouse Model
Source: Front Microbiol. 2022 Jul 5;13:906961. doi: 10.3389/fmicb.2022.906961 (PMC9294509; doi:10.3389/fmicb.2022.906961)
Supplement: Supplementary file 1 [file Data_Sheet_1.docx]

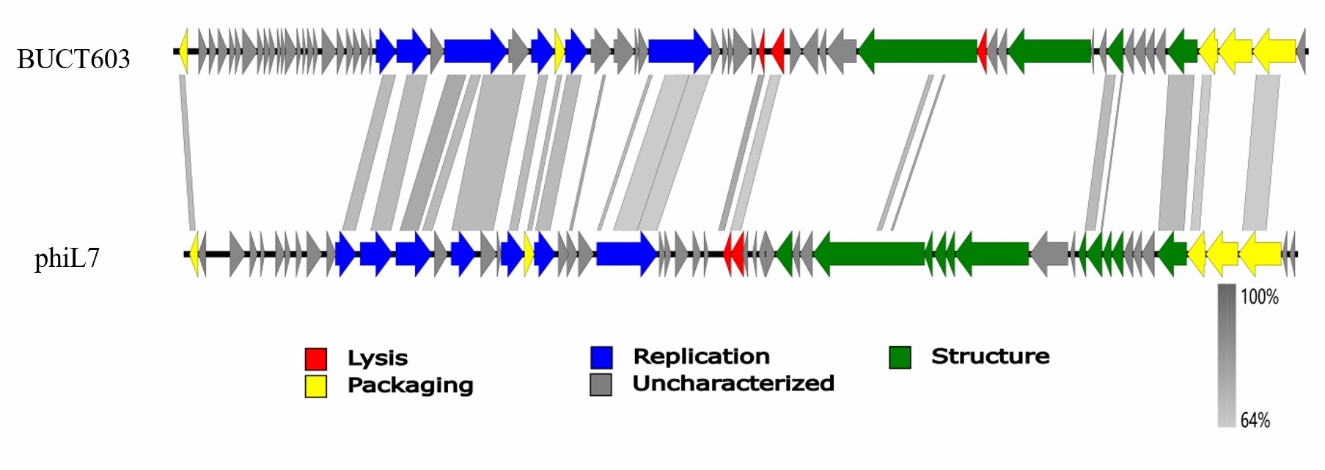


**Figure S1.** Schematic genomic alignment of the phage BUCT603 with Xanthomonas phage phiL7. Gray

shading indicates phage BUCT603 ORFs similarity sharing more than 60% with that of phage phiL7.


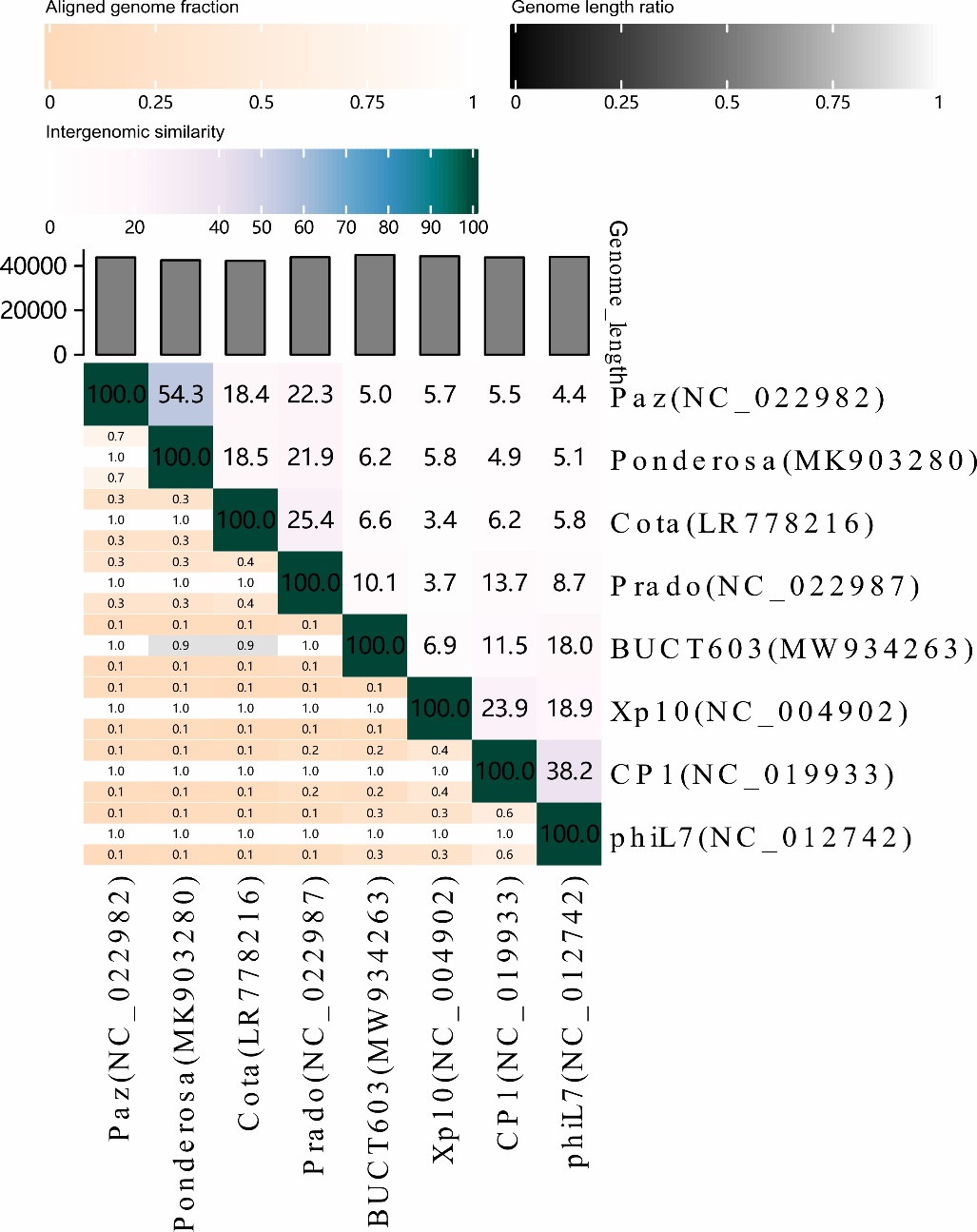


**Figure S2.** Percentage sequence similarity between phages calculated using VIDIRIC. The horizontal and vertical coordinates indicate the corresponding phage and Genebank number.


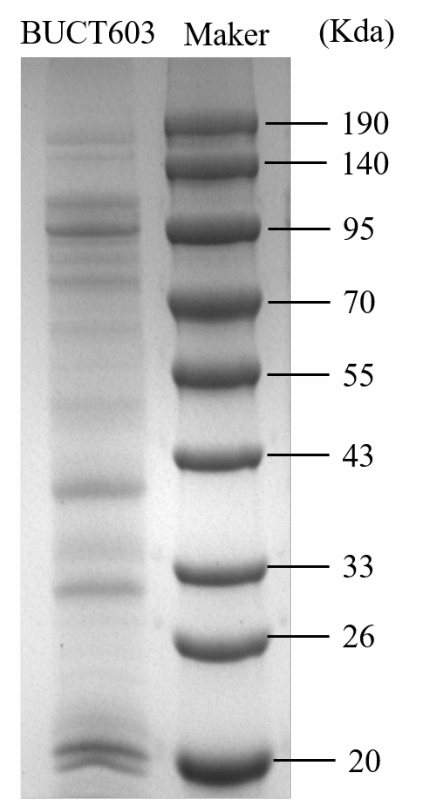


**Figure S3.** The SDS-PAGE analysis of phage BUCT603 structural proteins on 10% gel.

**Table S1.** Antibiotic sensitivity test results of 14 *S. maltophilia* strains.

| **Strains** | **Sulfamethoxazole** | **Ampicillin** | **Levofloxacin** | **Cefuroxime** | **Minocycline** | **Imipenem** | **Gentamicin** |
| --- | --- | --- | --- | --- | --- | --- | --- |
| SM35 | S | R | S | R | S | R | R |
| SM118 | S | R | S | R | S | R | R |
| SM209 | S | R | S | R | S | R | R |
| SM532 | S | R | R | R | S | R | R |
| SM548 | S | R | S | R | S | R | R |
| SM690 | S | R | S | R | S | R | R |
| SM824 | S | R | R | R | S | R | R |
| SM826 | R | R | S | R | S | R | R |
| SM992 | S | R | S | R | S | R | S |
| SM1207 | S | R | R | R | S | R | R |
| SM1209 | S | R | S | R | S | R | R |
| SM1284 | S | R | S | R | S | R | R |
| SM1785 | S | R | S | R | S | R | R |
| SM1786 | R | R | S | R | S | R | R |

“S ” susceptible “R” resistance

**Table S2**. Sequences of primers used in this work

| **Primer** | **Sequence (5′ - 3′)** |
| --- | --- |
| KAN-2FP-1 | ACCTACAACAAAGCTCTCATCAACC |
| KAN-2RP-1 | GCAATGTAACATCAGAGATTTTGAG |
| tonB-F | aagcttATGAACGTTCGCACCCCTG |
| tonB-R | ggatccTCAGAAGCGCTGGTTGTAC |
